# Sex offending among adolescents and young men with history of psychiatric inpatient care in adolescence

**DOI:** 10.1002/cbm.2236

**Published:** 2022-04-13

**Authors:** Riittakerttu Kaltiala, Timo Holttinen, Noora Ellonen

**Affiliations:** ^1^ Tampere University Faculty of Medicine and Health Technology Tampere Finland; ^2^ Department of Adolescent Psychiatry Tampere University Hospital Tampere Finland; ^3^ Tampere University, Faculty of Social Science Tampere Finland

**Keywords:** adolescent inpatients, mental disorders, register study, sex offending

## Abstract

**Background:**

Some mental disorders have been associated with increased likelihood of sexual offending in adolescents (and adults), but relevant studies tend to be of established sex offenders.

**Aims:**

To examine relationships between adolescent mental disorders and subsequent involvement in sex offending and to explore any predictive value of primary diagnoses for subsequent interpersonal offending, whether sexual or violent.

**Methods:**

We analyse national register‐based longitudinal data on males in Finland admitted for their first psychiatric inpatient treatment between the ages of 13–17 in the period 1980–2010 (*N* = 6749). Cox regression was used for the analysis of multivariate associations.

**Results:**

A subsequent criminal record for sex crime in the 10‐year follow up was rare among former child and adolescent psychiatric (CAP) inpatient males (1.5%). Having a subsequent criminal record for non‐sex‐related violent crime was more common (25%). Time to either sex crimes or non‐sex‐related violent crimes after a first CAP inpatient treatment was 3–4 years. Whilst the risk of committing non‐sex‐related violent crimes was elevated in all diagnostic groups compared to those with schizophrenia spectrum disorders, the risk of committing subsequent sex crimes was elevated only in the group with substance use, conduct or personality disorders. Among those with pre‐existing criminal history of sex crime, the risk of a subsequent criminal record for sex crime after CAP treatment was increased 11‐fold, but the risk for later non‐sex‐related violent crimes was not increased compared to the rest of the male adolescent CAP population.

**Conclusions and implications for practice:**

In this first longitudinal study of criminal convictions for sex offending after a period of inpatient psychiatric treatment as an adolescent such convictions were rare, but the difference in post discharge risk of further convictions for sexual offending and non‐sexual violent offending raises questions about whether more attention and specific treatment for aberrant sexual behaviours is needed for male adolescents with severe mental disorders.

During adolescence, discrepancy between physical, cognitive, and emotional maturation (Cacciatore et al., 2019; Steinberg, 2005) may increase the risk of engaging in risky, even non‐consensual sexual encounters, that are associated with both internalising and externalising mental disorders (Cacciatore et al., 2019; Kaltiala‐Heino et al., 2015; Savioja et al., 2015). Being subjected to non‐consensual sexual activity is a major risk factor for mental disorders (Fergusson et al., 1996; Perez‐Fuentes et al., 2013), but perpetrating it may also be associated with developmental problems and mental disorders. In addition to the general risk factors for delinquency, juvenile sex offenders often present with a history of subjection to sexual abuse, atypical sexual interests, social isolation, and internalising psychopathology (Seto & Lalumiere, 2010).

Some psychiatric and developmental conditions have been associated with increased likelihood of sexual offending in adolescents (and adults). These include autism spectrum disorders and mental retardation (Blasingame, 2018; Hofvander et al., 2019; Mogavero, 2016; Nixon et al., 2017; 't Hart‐Kerkhoffs et al., 2015) and severe conduct disorders/antisocial personality development (McCuish et al., 2015; Seto & Lalumiere, 2010). Up to two‐thirds of juvenile sex offenders meet the diagnostic criteria for some mental disorders, often comorbid, or display remarkable self‐rated psychopathology (Boonmann et al., 2015; Boonmann, Grisso, et al., 2016; Boonmann, Nelson, et al., 2016; 't Hart‐Kerkhoffs et al., 2015; Van Wijk et al., 2007).

Many severe mental disorders present with features with the potential to distort normative development towards consensual, mutually satisfactory intimate sexuality, among them anhedonia, deficits in impulse control, social anxiety or problems in social perception and communication. These may be particularly harmful during adolescence when sexuality undergoes decisive development. Studies on associations between juvenile sex offending and mental health have mainly been based on offender samples at various stages of the judicial process and have reported on psychopathology cross‐sectionally (Boonmann et al., 2015; Boonmann, Grisso, et al., 2016; Boonmann, Nelson, et al., 2016; Seto & Lalumiere, 2010; 't Hart‐Kerkhoffs et al., 2015; Van Wijk et al., 2007). Adult population studies have reported on mental disorders after sex crimes have been committed (Fazel et al., 2007, 2010). A gap in existing knowledge is that none of these studies have assessed subsequent involvement in sex offending among adolescents with severe mental disorders, taking into account all kinds of severe mental disorders in the same study and following up the adolescents into adulthood.

Our aim was to test for associations between severe mental disorders in adolescence and subsequent involvement in sex offending. More specifically we asked:

1. How commonly do male adolescents admitted to psychiatric inpatient care for the first time in their lives acquire a criminal record for sex offences within 10 years of the first psychiatric inpatient treatment?
2. For those who offend sexually, how soon after the index treatment does this occur?
3. Which of the primary diagnoses carries the greatest risk of subsequent sex offending?
4. Are any longitudinal associations between severe mental disorders and sex offences similar to or different from associations between severe mental disorders and non‐sex‐related violent crimes?

Finally, as our data extends over 3 decades, we took account of the historical context. The societal effects and the social meaning of being psychiatrically hospitalised in adolescence are likely to have changed over time (Neil & Sampson, 2021), also true of treatment (Holttinen et al., 2021), therefore we also asked:

5. Are there differences in the likelihood of subsequently acquiring a criminal record due to sex offences among male adolescents admitted to psychiatric inpatient care in the 1980s, 1990s and 2000s?

## 1 METHOD

### 1.1 Ethics

The study was approved by the ethics committee of Tampere University Hospital and appropriate permissions were granted by the National Institute of Health and Welfare and Statistics Finland.

### 1.2 Cohort identification

Young people admitted between 1980 and 2010 for their very first psychiatric inpatient treatment at ages 13–17 in Finland were identified from the Care Register for Health Care of the National Institute for Health and Welfare (Gissler & Haukka, 2004). Altogether 17, 112 adolescents so admitted were identified. Selection criteria for the present analyses were male sex and a primary (recorded as the first) diagnosis in the psychiatric (F), neurological (G) or social reasons (Z) categories (World Health Organization, 1992), yielding a total of 6749.

### 1.3 Procedure

Criminal history was obtained from the Register of Prosecutions, Sentences and Punishments maintained by Statistics Finland. These statistics include data on all sentences imposed and waived, and charges rejected by courts of first instance. In addition to the prosecutions at district courts and at courts of appeal acting as courts of first instance, the statistics also contain data on the summary penal fines imposed by the prosecutor and on petty fines imposed by the police, customs officials or border guard authorities. Register entries after the index admission were included for up to 10 years, with a census date of 31 December 2014. Inevitably, therefore, later admissions had less time at risk. While offences after the index admission constituted the outcome variable, register entries before the index admission were also recorded and used as a covariates. The age of criminal responsibility in Finland is 15 years.

### 1.4 Measures

A first admission to psychiatric inpatient care at age 13–17 years was taken as the index admission. For this, dates of admission and discharge, primary (first) diagnosis and the patient's sex and age were recorded.

In 1969–1986, the diagnoses were recorded according to ICD‐8, in 1987‐1995 according to ICD‐9 and since 1996 according to ICD‐10 (World Health Organization, 1965, 1977, 1992). All diagnoses were converted to the ICD‐10. Thus, the primary (first) diagnoses were grouped according to ICD‐10 (World Health Organization, 1992) as follows: F00–09, F70–79, F80–89 and G diagnoses (organic mental disorders, intellectual disability, developmental disorders and the few cases where the primary diagnosis recorded was not psychiatric but neurological); F20‐29 (schizophrenia group diagnoses); F30‐39 (mood (affective) disorders); F10–19, F60–69 and F90–92 (substance use, personality and conduct disorders); (F40–49, F59–59 and F93–99 (eating, anxiety and childhood onset emotional disorders); Z‐codes (factors influencing health status and contact with health services; no actual diagnosis; Table 1).

**TABLE 1:** Primary diagnoses among boys admitted to psychiatric inpatient care for the first time at ages 13–17 in 1980–2010 in Finland

	All %	Males only %
	(*n* = 16,842)	(*n* = 6749)
Organic, intellectual disability and developmental disorders (F00–09, F70–79, F80–89, G‐diagnoses)	3.0 (511/16,842)	4.9 (332/6749)
Schizophrenia group (F20–29)	11.5 (1932/16,842)	14.1 (952/6749)
Severe mood disorders (F30–39)	28.7 (4842/16,842)	19.0 (1284/6749)
Substance use, personality and conduct disorders (F10–19, F60–69, F90–92)	21.4 (3611/16,842)	29.4 (1985/6749)
Anxiety, eating and emotional disorders of childhood (F40–49, F59–59, F93–99)	31.9 (5301/16,842)	27.9 (1886/6749)
Z‐codes	3.8 (645/16,842)	4.6 (310/6749)

For criminal conduct, the date and title of the crime were recorded. The analyses were run separately for sex crimes (rape, coercion into sexual act, sexual abuse, sexual harassment, sexual abuse of a child, abuse of a victim of sexual trade, purchase of sexual services from an underaged person, solicitation of a child for sexual purposes, including attempted and aggravated forms) and for non‐sex‐related violent crimes (murder, attempted murder, manslaughter, attempted manslaughter, assault, aggravated assault, robbery, robbery, arson, fire‐setting).

### 1.5 Statistical analyses

First, cross‐tabulations with chi‐square statistics were used. Crime outcome, (a) sex and (b) non‐sex‐related violent crime within 10 years of discharge, was compared between those in early (13–14 years old) and middle (15–17 years old) adolescence at index admission, between those with index admission in the 1980s, 1990s and 2000s, between those with and without an entry in the crime register for sex/non‐sex‐related violent crime before the index admission and between the diagnostic groups. Incidence rates for (a) sex and (b) non‐sex‐related violent crime per 100 person‐years were calculated taking into account the competing risks and attrition within the 10‐year time frame; each person was followed up until the first recorded occurrence of the outcome of interest or to a maximum of 10 years, or to death, whichever occurred first. Incidence rates were likewise compared between the diagnostic groups.

Multivariate associations were studied using Cox's regression to assess the relationship between survival time and covariates. Hazard ratios (95% confidence intervals) for (a) sex crime and b) violent crime are given according to diagnostic group, sex, age group and decade of index admission, also controlling for whether or not the subject had a crime register entry before the index admission. Mean (SD) and median times (interquartile ranges) to defined (a) sex crime and (b) violent crime after discharge from the index admission are given for diagnostic groups. Hazard curves are presented. Due to the large data size, the cut‐off for statistical significance is set at *p* < 0.001.

## 2 RESULTS

### 2.1 Sex crimes

Of all the former male inpatients, 103 (1.5%; incidence rate 0.2) had committed sex crimes during follow‐up. Acquiring a criminal record for sex crimes was equally common among those with index admission in early (13–14 years) and middle adolescence (15–17 years) and among those admitted in 1980‐89, 1990–1999 and 2000–2010. Of those with no criminal record for sex crimes before the index admission (*n* = 6721), 1.5% (98) had committed sex crimes after the index admission but, among the 28 having a prior record for sex crimes, 18% (5), a significant difference (*p* < 0.0001). Incidence rates (IR) were 0.2 and 2.5 respectively (*p* < 0.0001). By contrast, of those with no criminal record for sex crimes, 25% (1670/6721; IR 3.5) subsequently acquired a record for non‐sex‐related violent crimes compared with 32% (9/28) of those with a prior record for sex crimes (IR 4.8; *p* = 0.37).

A criminal record for sex crimes after the index admission was most common among those with primary diagnoses of substance use, personality or conduct disorder (F10–19, F60–69, F90–92), followed by those whose index admission had been recorded with a Z‐code. It was least common in those with mood disorders (F30–39; Table 2)

**TABLE 2:** Sex crime and non‐sex‐related violent crime by primary diagnosis during follow‐up of a maximum 10 years after index admission among boys admitted to psychiatric inpatient treatment for the first time at ages 13–17 between 1980 and 2010 in Finland. (% (*n*/*N*))

	F00‐09, F70‐79, F80‐89,G‐diagnoses	F20–29	F30–39	F10–19, F60–69, F90–92	F40–49, F50–59, F93–99	Z‐code	*p*
Sex crime^a^	1.2 (4/332)	0.9 (9/952)	0.8 (10/1284)	2.8 (56/1985)	0.9 (17/1886)	2.3 (7/310)	<0.001
Incidence rate	0.1	0.1	0.1	0.3	0.1	0.3	
Non‐sex‐related violent crime^b^	10.2 (34/332)	12.0 (114/952)	16.8 (216/1284)	41.6 (825/1985)	21.5 (406/1886)	27.1 (84/310)	<0.001
Incidence rate	1.3	1.5	2.3	6.7	3.0	3.7	

^a^ In pairwise comparisons, the group (F10‐19, F60‐69, f90‐92) differed statistically significantly at level *p* < 0.001 from all the other groups except Z‐code.

^b^ In pairwise comparisons, the group (F10‐19, F60‐69, F90‐92) different statistically significantly at level *p* < 0.001 from all the other groups; the Z‐code group differed from all the other groups, and the group (F40‐49, F50‐59, f90‐92) further from (F20‐29) and (F00‐09, F70‐79, F80‐89, G).

Mean (SD) time to the first sex crime was 4.01 (2.67) years and median (Q1:Q3) time was 3.44 (1.94; 5.85). Shortest times were seen among those who had a Z‐code as the primary diagnosis at index admission, but there was considerable overlap of interquartile ranges between diagnostic groups, suggesting that the differences in median times were not statistically significant (Table 3).

**TABLE 3:** Mean(sd) and median (IQR, Q1; Q3) time in years to first sex crime and first non‐sex‐related violent crime after discharge from index admission to a child and adolescent psychiatric inpatient unit

Diagnostic groups							Total
	F00–09 F70–89 F80–89 G‐diagnoses (*n* = 332)	F20–29 (*n* = 952)	F30–39 (*n* = 1284)	F10–19 F60–69 F90–92 (*n* = 1985)	F40–48 F50‐59 F93‐99 (*n* = 1886)	Z‐code (*n* = 310)	
Time to (first) sex crime (years)							
Mean(SD)^a^	4.11 (2.26)	4.85 (3.02)	3.66 (2.15)	3.83 (2.51)	4.55 (3.30)	3.53 (3.14)	4.01 (2.67)
Median	4.27	4.05	3.41	3.40	2.87	2.69	3.44
IQR	2.91	5.15	3.41	3.42	5.68	3.38	3,91
Q1	2.66	3.06	2.16	1.94	2.01	1.33	1.94
Q3	5.57	8.21	5.57	5.36	7.69	4.71	5.85
Time to (first) non‐sex violent crime (years)							
Mean (SD)^b^	3.89 (2.38)	3.51 (2.55)	3.55 (2.51)	3.20 (2.42)	3.45 (2.53)	3.59 (2.58)	3.36 (2.48)
Median	3.56	2.95	3.17	2.79	2.82	3.22	2.89
IQR	3.26	4.11	3.86	3.52	3.48	3.90	3.58
Q1	2.00	1.33	1.47	1.23	1.57	1.36	1.35
Q3	5.76	5.44	5.33	4.75	5.02	5.26	4.93

^a^ No statistically significant difference between mean times (ANOVA *p* = 0.83).

^b^ No statistically significant difference between mean times (ANOVA *p* = 0.16).

### 2.2 Non‐sex‐related violent crimes

Non‐sex‐related violent crime after the index admission was recorded for 1679 (25%; IR 3.5) of the former CAP inpatients, and equally commonly for those with their index admission in early (13–14 years) or in middle adolescence (15–17 years). The proportion of those obtaining a subsequent criminal record for non‐sex‐related violent crime was smallest but the absolute number greatest among those admitted during the latest decade (2000–10; 394 (30%), IR 3.8 versus 483 (30.7%), IR 3.6 versus 802 (21%), IR 3.2; *p* < 0.001). Having a criminal record for non‐sexual violence prior to admission was associated with greater risk of such crimes after admission (no prior violent crime 22% (1409/6313) versus prior violent crime 62% (270/436); *p* < 0.0001). Incidence rates were 3.1 and 13.1 respectively (*p* < 0.0001). Post discharge sex crimes were also more likely among those with a prior non‐sexual violence criminal (3.4% (15/436) versus 1.4% (88/6313; IR 0.4: 0.2; *p* = 0.001).

Record of non‐sex‐related violent crime after index admission was most common among those with primary diagnoses of substance use, personality or conduct disorder (F10–19, F60–69, F90–92), followed by those for whom a Z‐code was given as the reason for the index admission. The smallest proportion of those subsequently acquiring a criminal record was seen among those with schizophrenia group diagnoses (F20–29) (Table 2).

Mean (SD) time to the first non‐sex‐related violent crime was 3.36 (SD 2.48) years, and median (Q1:Q3) time was 2.89 (1.35; 4.93). Shortest times were seen among those with primary diagnoses at index admission of substance abuse/personality disorder/conduct disorder group (F10‐19, F60‐69 or F90‐92), but there was considerable overlap of interquartile ranges between diagnostic groups suggesting that the differences in median times were not statistically significant (Table 3).

### 2.3 Multivariate analyses

In the multivariate models, all the variables of interest were entered simultaneously. Findings are reported adjusted for time at risk.

**FIGURE 1:**
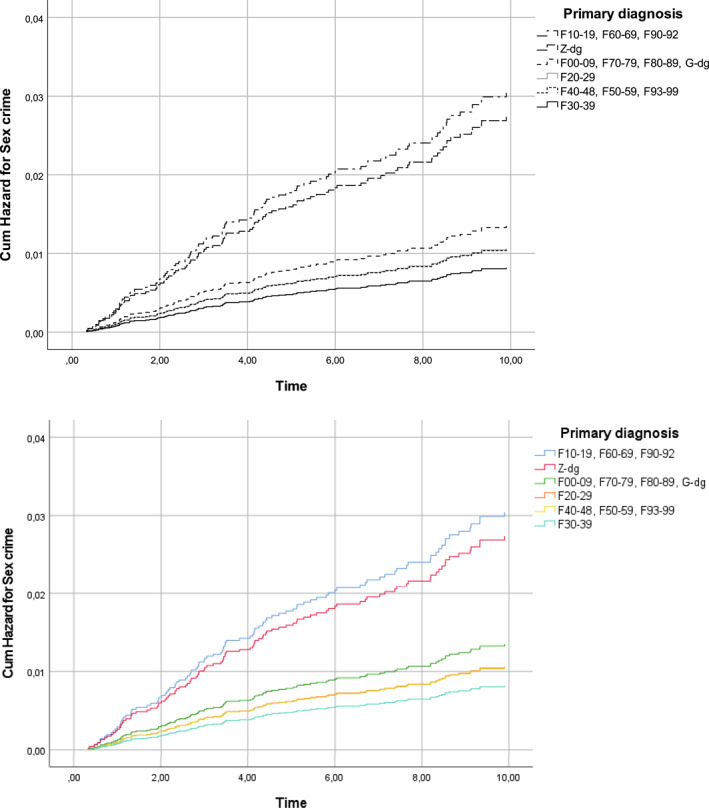
Cumulative hazard for sex crime according to primary diagnosis among boys who had been admitted to a child and adolescent psychiatric inpatient unit in Finland

First, with the schizophrenia group of diagnoses (F20‐29) as the reference, Table 4 shows that the diagnoses in the substance use, personality and conduct disorder groups (F10‐19, F60‐69, F90‐92) were associated with between two and four times the risk of acquiring a criminal record for sexual offences post‐admission. The risk was smaller among those admitted in the 1990s than among those admitted in the other decades of the study period. Table 4 also confirms that having had a criminal record for sex crime before the index admission increased the risk of post‐discharge sex crime 11.4‐fold (Table 4). Figure 1 showed the increasing risk over time according to diagnosis.

**TABLE 4:** Hazard ratios (95% confidence intervals) for acquiring a criminal record for sex crime and for non‐sex‐related violent crime within a maximum 10 years after index admission among boys initially admitted for psychiatric treatment at ages 13–17 between 1980 and 2010

	Model 1: Sex crime		Model 2: Non‐sex violent crime	
	HR (95% CI)	*p*	HR (95% CI)	*p*
Age				
13–14	ref.		ref.	
15–17	1.3 (0.8–1.9)	0.3	1.0 (0.9–1.2)	0.6
Year of index admission				
2000–2010	ref.		ref.	
1990–1999	0.5 (0.3–0.8)	0.01	1.3 (1.1–1.5)	<0.001
1980–1989	0.6 (0.4–1.1)	0.07	1.3 (1.2–1.5)	<0.001
Primary diagnosis at index admission				
F20–29	ref.		ref.	
F00–09, F70–79, F80–89, G	1.3 (0.4–4.2)	0.7	0.9 (0.6–1.4)	0.7
F30–39	0.8 (0.3–1.9)	0.6	1.7 (1.4–2.1)	<0.001
F40–49, F50–5, F93–99	1.0 (0.4–2.2)	1.0	2.0 (1.6–2.5)	<0.001
Z‐code	2.6 (0.9–7.0)	0.06	2.5 (1.9–3.3)	<0.001
F10–19, F60–69, F90–92	2.9 (1.4–5.9)	0.004	4.1 (3.4–5.0)	<0.001
Registered sex (model 1)/non‐sex‐related violent (model 2) crime before index admission				
No	ref.		ref.	
Yes	11.4 (4.6–28.6)	<0.001	3.0 (2.6–3.5)	<0.001

*Note*: HR, Hazard ratio; CI, Confidence interval.

**FIGURE 2:**
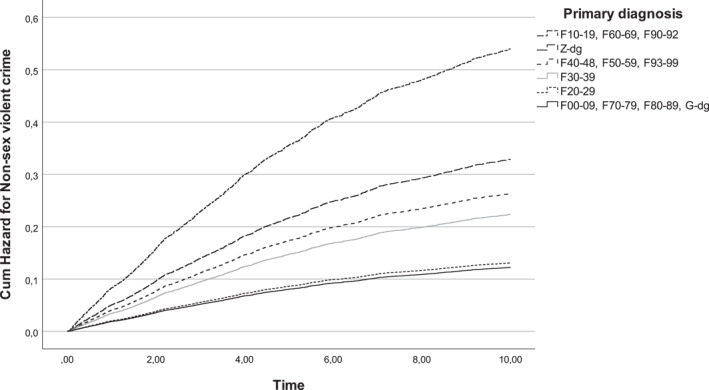
Cumulative hazard for non‐sex‐related violent crime according to primary diagnosis among boys who had been admitted to a child and adolescent psychiatric inpatient unit in Finland

The risk of acquiring a record for subsequent non‐sex‐related violent crime was increased in all other diagnostic groups studied relative to those with schizophrenia group disorders (F20‐29), except for those with organic, intellectual and developmental disorders (F00‐09, F70‐79, F80‐89). Compared to those admitted in the 2000s, the 1980–1999 admission cohort had a higher risk of non‐sex‐related violent crime. A pre‐admission history of non‐sex‐related violent crime was associated with three times the risk of post‐discharge non‐sex elated violent offending (Table 4; Figure 2).

## 3 DISCUSSION

Criminal conviction for sex‐crimes by adolescents or young men during the 10 years following discharge from a child and adolescent psychiatric inpatient facility was rare, and much less common than a record for non‐sex‐related violent crime. This concurs with the known prevalence of these types of crimes in general population (Danielsson, 2019). Mean and median times to both sex crimes and non‐sex‐related violent crimes after a person's first CAP inpatient treatment were 3–4 years, meaning that a criminal record was on average already acquired during the late adolescent years.

While the risk for committing non‐sex‐related violent crime was increased in all diagnostic groups compared with those with schizophrenia spectrum disorders, the risk for sex crimes was only statistically significantly increased in the group comprising substance use, conduct and personality disorders. Earlier research has also linked both sex and non‐sex‐related violent crimes with antisocial development typical for substance use, conduct and personality disorder (Fanniff et al., 2017; McCuish et al., 2015; Seto & Lalumiere, 2010).

First‐time admitted adolescent psychiatric patients with schizophrenia spectrum diagnoses were at lowest risk for subsequently committing both sex and non‐sex‐related violent crimes, and this also holds true for non‐violent crimes (Kaltiala et al., 2021). Schizophrenia group psychoses have earlier been associated with elevated risk of violent behaviours in both adolescents and adults (Gammelgard et al., 2008; Hachtel et al., 2018; Naudts & Hodgins, 2006; Taylor, 2008; Whiting et al., 2020), and also with sex crimes in adult samples (Fazel et al., 2007, 2010; Langstrom et al., 2004). Patients with early onset schizophrenia often have the most severe course of the disorder (Schimmelmann et al., 2013), which may also include an increased risk for violent behaviour. This may be balanced by early onset schizophrenia spectrum disorders being likely to be the most severe, resulting in most intensive care, which may reduce the risk of committing crimes in the community. Violent behaviours may occur in care institutions (Kaltiala‐Heino et al., 2013, 2014) but, when do, do not necessarily enter the official crime statistics.

Neither intellectual disability nor autism stood out as being associated with increased risk for sex offending. This need not necessarily contradict earlier findings suggesting associations between these and sex offending (Mogavero, 2016; Nixon et al., 2017). People with intellectual disabilities are primarily treated within their own specialised service system in Finland. Autism in adolescents without intellectual deficit was likely not recognized and therefore not recorded as a diagnosis before the 2000s (Turunen et al., 2010).

**FIGURE 3:**
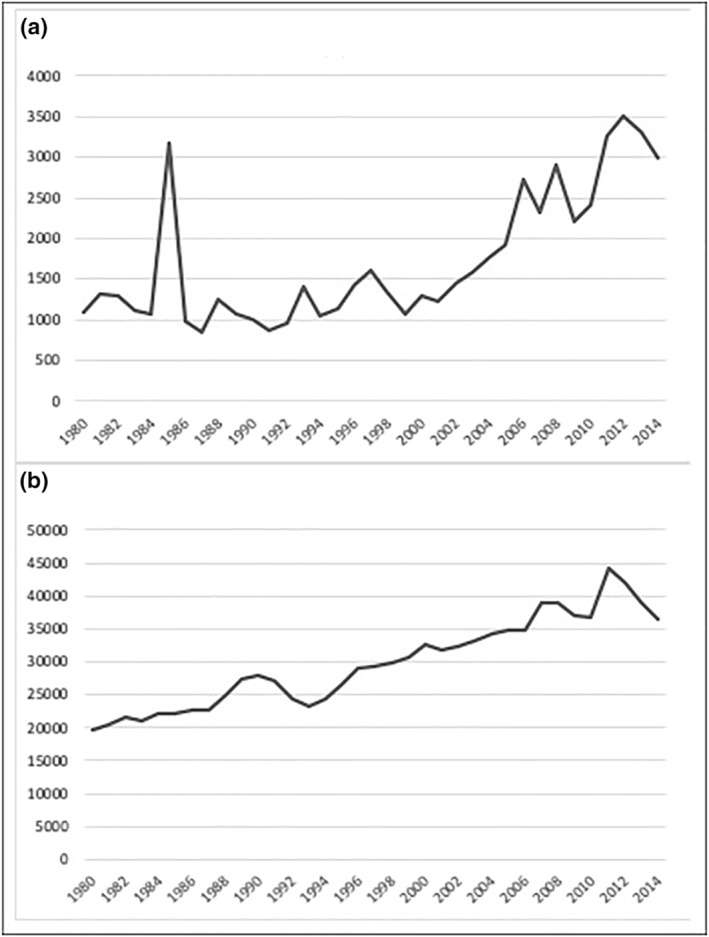
Sex crimes (a) and non‐sex‐related violent crimes (b) registered by police in Finland 1980–2014, based on data by Statistics Finland

Subsequent sex crime was more common and subsequent non‐sex‐related violent crime less common among those admitted in the latest decade, despite the shorter follow‐up time for those admitted after 2005. This may partially reflect societal changes in the reporting of sexual assaults to the police. Apart from a single peak in 1985, the number of sex crimes reported to the police remained relatively stable from 1980 to 2000 and thereafter increased three‐fold to 2014 (Figure 3).

When there was already a record for sex crimes before the index admission, the risk for subsequent entry in the criminal record for sex crime was increased 11‐fold, but the risk for later non‐sex‐related violent crimes was not increased. The proportion of sexual reoffenders in this CAP population was also greater than that suggested among juvenile sex offender populations (Fanniff et al., 2017; Lussier & Blockland, 2014; McCann & Lussier, 2008; Waite et al., 2005). The risk for subsequent sexual offending by prior non‐sex‐related violent crime was much smaller.

### 3.1 Clinical implications

It is unfortunately not known if sexual offending was specifically addressed in the inpatient care of those with prior crime record. Cognitive, behavioural and multidimensional family therapies focussing on deviant sexual arousal, cognitive distortions and rationalizations predisposing to offending behaviour, victim empathy and social skills, family relationships and appropriate parenting and personal trauma are recommended for adolescent sex offenders (Thibaut et al., 2016). According to the risk‐needs‐responsivity principles model of offender treatment (Andrews & Bonta, 2010; Ter Beek et al., 2018), treatment and monitoring should be the more intensive the greater the risk or number of needs. Adolescents with severe psychiatric disorders and sex offending histories are likely to represent a group with complex needs. In inpatient and subsequent community care, criminogenic needs should be addressed among those already having a criminal record.

#### 3.1.1 Methodological considerations

The study was based on registers containing mandatory reports of all incidents of interest by hospitals and criminal justice agencies. The uniquely large data comprised all those initially admitted to psychiatric inpatient care at ages 13–17 from 3 decades. The large and comprehensive data is a strength of this study.

The diagnoses were recorded as set by the treating physicians in the hospitals. Earlier studies have shown that in Finland psychiatric diagnostic work is very reliable in psychiatric inpatient care (Isohanni et al., 1997; Pihlajamaa et al., 2008).

A limitation of the study is that we could not include any other clinical characteristics of the CAP patients. Further research is needed to evaluate in more detail, for example, what symptom profiles are predictive of subsequent sex offending.

## CONFLICT OF INTEREST

None.

## Data Availability

The data that support the findings of this study is available from Findata. Restrictions apply to the availability of these data, which were used under license for this study. Data are available from the authors if permission is granted from Findata.
